# Emerging Roles of Aldehyde Dehydrogenase Isoforms in Anti-cancer Therapy Resistance

**DOI:** 10.3389/fmed.2022.795762

**Published:** 2022-03-01

**Authors:** Michele Zanoni, Sara Bravaccini, Francesco Fabbri, Chiara Arienti

**Affiliations:** Biosciences Laboratory,IRCCS Istituto Romagnolo per lo Studio dei Tumori (IRST) “Dino Amadori”, Meldola, Italy

**Keywords:** aldehyde dehydrogenase, cancer stem cell, double strand brakes (DSB), therapeutic resistance, immunosuppression

## Abstract

Aldehyde dehydrogenases (ALDHs) are a family of detoxifying enzymes often upregulated in cancer cells and associated with therapeutic resistance. In humans, the ALDH family comprises 19 isoenzymes active in the majority of mammalian tissues. Each ALDH isoform has a specific differential expression pattern and most of them have individual functional roles in cancer. ALDHs are overexpressed in subpopulations of cancer cells with stem-like features, where they are involved in several processes including cellular proliferation, differentiation, detoxification and survival, participating in lipids and amino acid metabolism and retinoic acid synthesis. In particular, ALDH enzymes protect cancer cells by metabolizing toxic aldehydes in less reactive and more soluble carboxylic acids. High metabolic activity as well as conventional anticancer therapies contribute to aldehyde accumulation, leading to DNA double strand breaks (DSB) through the generation of reactive oxygen species (ROS) and lipid peroxidation. ALDH overexpression is crucial not only for the survival of cancer stem cells but can also affect immune cells of the tumour microenvironment (TME). The reduction of ROS amount and the increase in retinoic acid signaling impairs immunogenic cell death (ICD) inducing the activation and stability of immunosuppressive regulatory T cells (Tregs). Dissecting the role of ALDH specific isoforms in the TME can open new scenarios in the cancer treatment. In this review, we summarize the current knowledge about the role of ALDH isoforms in solid tumors, in particular in association with therapy-resistance.

## Introduction

Aldehyde dehydrogenases are a family of NADP^+^-dependent enzymes that participate in several physiological and biosynthetic processes, catalyzing the oxidation of toxic aldehydes into carboxylic acids and participating in the synthesis of molecules involved in cell differentiation, proliferation and survival as retinoic acid (RA), γ-aminobutyric acid, and betaine (1). Aldehydes are toxic and highly reactive compounds that are present in the environment ubiquitously and can be uptaken by the organisms through air, food, and water (2). Alternatively, they are generated as intermediates from pathophysiological processes such as metabolism of amino acids, carbohydrates, vitamins and lipid peroxidation (3). In cancer, oxidative damage generated from conventional treatment as chemo and radiotherapy are mainly related to reactive oxygen species (ROS) production, which, in turn, results in further accumulation of aldehydes (3). The toxicity of these compounds is mainly related to their ability to covalently modify several cellular macromolecules including nucleic acids, amino acids and lipids inducing DNA damage, apoptosis and impairing cellular homeostasis and mitochondrial respiration (4, 5). In humans, ALDH superfamily comprises 19 isoenzymes divided in 11 families (6) localized in different cellular compartments including nucleus, cytoplasm, mitochondria, and endoplasmic reticulum (7). ALDH enzymes are basically present in all mammalian tissues, with the highest levels in liver and kidney (7). Each ALDH enzyme has one or more specific substrates and currently several ALDH inhibitors are at different stages of development. Altered ALDH activity is associated with several disorders and syndromes as the Sjogren–Larsson syndrome, Fanconi anemia, g-hydroxybutyric aciduria, dermatitis, pyridoxine-dependent seizures, type II hyperprolinemia, Alzheimer’s and Parkinson’s diseases, and different cancer types (Table 1) (3). In the present review, we summarize the current state of art on the role of ALDH isoenzymes in solid tumors, with a particular focus on their involvement in cancer stem cells (CSCs), therapy resistance and immune suppression. Finally, we describe the translational applications of ALDHs as potential innovative anticancer therapeutic targets.

**TABLE 1:** Human aldehyde dehydrogenase isoenzymes function and cancer.

ALDH enzymes	Subcellular localization	Chromosomal localization	Functional activity	Types of cancer
ALDH1A1	Cytosol	9q21.13	Oxidation of retinaldehyde to retinoic acid; Oxidation of acetaldehyde, and lipid peroxidation-derived aldehydes	Thyroid, liver, cervical, pancreatic, melanoma, prostate, endometrial, breast, colorectal, renal
ALDH1A2	Cytosol	15q21.3	Oxidation of retinaldehyde to retinoic acid	Ovarin, prostate, leukemia, thyroid, liver, cervical, pancreatic, melanoma, endometrial, breast, colorectal, renal, glioma, head and neck, carcinoid
ALDH1A3/ALDH6	Cytosol	15q26.3	Oxidation of retinaldehyde to retinoic acid	Pancreatic, ovarian, breast, glioma, melanoma, prostate, testis, liver, colorectal, urothelial, head and neck, carcinoid, cervical, renal, stomach, endometrial
ALDH1B1/ALDH5	Mitochondria	9p13.1	Oxidation of acetaldehyde and lipid peroxidation-derived aldehydes	colon, pancreatic, osteosarcoma, thyroid, lung, head and neck, stomach, liver, carcinoid, renal, prostate, testis, breast, ovarian, melanoma, lymphoma, urothelial, endometrial, skin, glioma, cervical
ALDH1L1/FDH	Cytosol	3q21.3	Conversion of 10-formyltetrahydrofolate (10-formyl-THF) in tetrahydrofolate	Liver, kidney, pancreatic, endometrial, urothelial, prostate, thyroid
ALDH1L2/mtFDH	Mitochondria	12q23.3	Conversion of 10-formyltetrahydrofolate (10-formyl-THF) in tetrahydrofolate	Prostate, colorectal, breast, thyroid, melanoma, pancreatic, carcinoid, glioma, stomach, urothelial, ovarian, cervical
ALDH2	Mitochondria	12q24.12	Oxidation of alcohol and lipid peroxidation derived acetaldehyde, 4-hydroxy-2-non-enal (4-HNE), and malondialdehyde (MDA)	Melanomas, lung, pancreatic, gliomas, colorectal, breast, ovarian, stomach
ALDH3A1	Plasma membrane Cytosol	17p11.2	Oxidation of aromatic aldehydes derived from alcohol, corticosteroids, biogenic amines, neurotransmitters metabolisms and from lipid peroxidation	Lung, liver, pancreatic, esophagus, skin, breast, ovarian, stomach, head and neck,
ALDH3A2/FALDH	Endoplasmic reticulum Peroxisomes	17p11.2	Oxidation of long-chain aliphatic-aldehydes into fatty acids	Malignant tissues were in general negative. Hepatocellular, thyroid
ALDH3B1/ALDH7	Plasma membrane Cytosol	11q13.2	Oxidation of long-chain lipid-derived aldehydes and benzaldehyde.	Lung, head and neck, liver, skin, esophagus, stomach, ovarian, renal, testis, colorectal, pancreatic
ALDH3B2/ALDH8	Lipid droplet	11q13.2	Oxidation of long-chain aldehydes into non-toxic fatty acids.	Renal, malignant melanomas, pancreatic, colorectal
ALDH4A1/P5CD	Mitochondria	1p36.13	Oxidation of glutamic gamma-semialdehyde, succinic, glutaric and adipic semialdehydes in glutamate.	In all cancers except lymphomas
ALDH5A1/SSADH	Mitochondria	6p22.3	Conversion of succinic-semialdehydes in succinate. Degradation of gamma-aminobutyric acid (GABA) neurotransmitter.	In all cancers
ALDH6A1/MMSDH	Mitochondria	14q24.3	Oxidative decarboxylation of malonate and methylmalonate semialdehydes to acetyl- and propionyl-CoA. Involved in valine and pyrimidine metabolism.	Thyroid, glioma, melanoma, renal, lung, head and neck, stomach, ovarian, breast, prostate, bladder, colorectal, pancreatic
ALDH7A1/EPD	Nucleus Cytosol Mitochondria	5q23.2	Metabolization of betaine aldehyde to betaine. Oxidation of lipid peroxidation-derived aldehydes. Involved in lysine catabolism.	Thyroid, glioma, renal, lung, stomach, breast, prostate, liver, colorectal, pancreatic, urothelial, testis
ALDH8A1	Cytosol	6q23.3	Oxidation of 2-aminomuconic semialdehyde to 2-aminomuconate in the kynurenine pathway of tryptophan catabolism.	Liver
ALDH9A1/ALDH4	Cytosol	1q24.1	Oxidation of gamma-aminobutyraldehyde and other amino aldehydes.	Thyroid, glioma, melanoma, renal, lung, head and neck, stomach, ovarian, breast, prostate, bladder, colorectal, pancreatic, cervical, urothelial, testis
ALDH16A1	Plasma membrane Cytosol	19q13.33	Not fully discovered. Involved in mast syndrome and gout.	Thyroid, glioma, melanoma, lung, head and neck, stomach, ovarian, breast, lymphoma, colorectal, pancreatic, cervical, urothelial, testis
ALDH18A1/P5CS	Mitochondria	10q24.1	Conversion of glutamate to glutamate 5-semialdehyde, an intermediate in the biosynthesis of proline, ornithine and arginine.	Colorectal, stomach, carcinoid, pancreatic, ovarian, thyroid, breast, liver, melanoma, cervical, testis, lymphoma, endometrial, head and neck, urothelial, lung, glioma, renal, prostate, skin

*Data derived from https://www.uniprot.org, https://www.proteinatlas.org, and https://www.ensembl.org/index.html databases.*

## Function of Aldehyde Dehydrogenases

**FIGURE 1:**
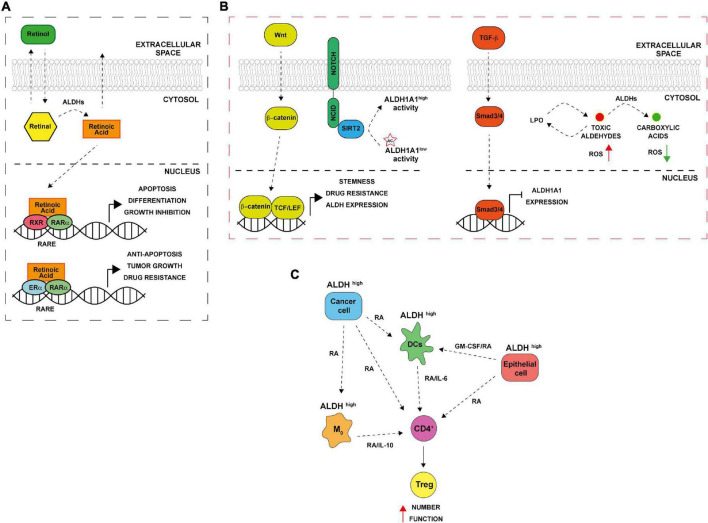
Aldehyde dehydrogenases (ALDHs) functional mechanisms. **(A)** ALDH in normal and cancer cells. Retinol (vitamin A) absorbed by normal and cancer cells is converted to retinal in the cytosol. ALDH enzymes oxidize retinal to retinoic acid (RA). RA can be secreted and diffused into neighboring cells or translocated into the nucleus where it binds to dimers of the retinoic acid receptor (RAR) and retinoic X receptor (RXR), inducing the expression of its downstream target genes that are involved in differentiation, apoptosis, and growth inhibition. In ER positive cancer cells, alternative RA signaling induces the expression of genes related to cell proliferation, stemness, tumor growth and anti-apoptosis. RARE (retinoic acid response elements). **(B)** ALDH and CSCs. The Wnt pathway directly regulates ALDH1A1 through β-catenin/TCF-dependent transcription, promoting drug resistance and stemness. TGF-β downregulating ALDH1A1 in Smad4- dependent manner. NOTCH signaling drives ALDH proteins deacetylation by SIRT2. In addition, ALDHs promote the oxidation of toxic reactive aldehydes into less toxic carboxylic acids limiting reactive oxygen species (ROS) production and lipid peroxidation. **(C)** ALDH and immune tolerance. Cancer cells with high ALDH levels release factors including RA that in turns increase ALDH levels in several cells of the TME including macrophages (M_0_), dendritic cells (DCs) and normal epithelial cells. The consequent RA production enhances Treg differentiation from CD4 + T cells; increasing their activity thus leading to immune tolerance.

The ALDH family is involved in several biological processes essential for cell survival along with cell protection. Besides, their role in detoxifying aldehydes, ALDHs perform other important functions including directly absorbing ultraviolet light, scavenging hydroxyl radicals *via* cysteine and methionine sulfhydryl groups, serving as binding proteins for various molecules (e.g., androgen and cholesterol) and also play important antioxidant roles by producing NAD(P)H (1, 7–9). Cytosolic class I ALDH enzymes function in RA cell signaling *via* RA production by oxidation of both all-*trans*-retinal and 9-*cis*-retinal to all-*trans*-retinoic acid and 9-*cis*-retinoic acid (Figure 1A) (10–12). Retinol or vitamin A, which is absorbed by cells, is first oxidized to retinal, then, retinal is irreversibly oxidized to RA by cytosolic ALDH1 isozymes (ALDH1A1, ALDH1A2, and ALDH1A3). The latter reaction is a tightly regulated tissue specific process. The lipophilic RA produced can function in paracrine or endocrine manner by diffusing into neighboring cells or the nucleus. Inside the nucleus, RA bind retinoic acid receptor α (RARα) and retinoic X receptor (RXR) transcription factors to induce transcriptional activity of target genes involved in development, apoptosis, and differentiation (Figure 1A) (10, 13, 14). In cancer, when RA binds to RARα, alternative transcriptional partners, as estrogen receptor α (ERα), are recruited into the nucleus, thus promoting cell proliferation, drug resistance, and inhibition of apoptosis through the activation of c-MYC, cyclin D1 and ALDH1A1 itself (Figure 1A) (15, 16). Additionally, both class I and II ALDH enzymes metabolize toxic aldehydes to reduce the ROS levels, and regulate self -renewal, cell differentiation and chemoresistance capabilities of CSCs and immune evasion by several pathways (Figures 1B,C) (10, 17). Indeed, the ALDHs participate in RA synthesis both in normal stem cells (SCs) and CSCs and provide a survival advantage to cancer cells leading to cancer progression and therapy resistance.

## Aldehyde Dehydrogenases in Normal and Cancer Stem Cells

About two decades ago it was first demonstrated that hematopoietic stem cells are enriched with ALDH1 enzyme (18). In particular, isoenzymes such as ALDH1A1 and ALDH3A1 play a functional role in normal stem cells for self-protection, expansion and differentiation (19). Furthermore, high cytosolic expression levels of ALDHs have been found not only in hematopoietic stem cells but also in several other types of normal stem cells, for instance, in mammary (20, 21), intestinal (22), neural (23), and prostate (24) tissues. In the overlap gene profiles of different stem cell populations, ALDH7A1, known as antiquitin, and ALDH2 were identified, consequently. Besides normal tissue stem cells, malignant stem cells are reported to express ALDHs and show high activity of these isozymes (19, 21). ALDH1A and 3A1 family (ALDH1A, ALDH1A2, ALDH1A3, and ALDH3A1) are essential in protecting SCs against toxic endogenous and exogenous aldehydes and for SCs’ ability to differentiate. These two isoenzymes’ families are largely described also in cancer, being mainly involved in drug resistance and in metastatic process (25, 26). Among them, the isoform ALDH1A3 is highly expressed in several tumors, including pancreatic cancer, gliomas and ovarian cancer but not expressed in the neighborhood no neoplastic cells (27). ALDH1A3 is overexpressed in CSCs characterized by a marked drug resistance and the capacity to promote self-renewal, clonogenic growth and tumor-initiating capacity. Indeed, post-transcriptional regulation of ALDH1A3 by autophagy induced by high concentration-temozolomide treatment was reported in glioblastoma cell lines (28). Attenuation of ALDH1A3 expression by RNA interference (RNAi) significantly suppressed cell proliferation, reduced the number of cancer cells that persisted after anticancer treatment and interfered with tumor growth in a mouse xenograft model (29). It has been shown that also other ALDH enzymes are activated both in SCs and CSCs, including ALDH2*2 (with an association between alcoholism and alcohol-induced cancer risk), ALDH4A1 (activated through p53 and DNA damage), and ALDH7A1 (putatively involved in the regulation of cell cycle) (1). Furthermore, high ALDH activity, combined with high telomerase activity and the presence of ATP-binding cassette (ABC) transporter G2 (ABCG2), is considered as a universal stem cell marker (30, 31). Indeed, a specific and widely used assay to measure ALDH activity called “aldefluor assay” has been originally used for the isolation of hematopoietic stem cells and now is commonly used for the CSCs isolation in many cancers (21, 32). In the beginning, high ALDH activity in CSCs was exclusively attributed to ALDH1A1 isozyme, while in recent years this high activity has been associated to other isoforms too, for instance, ALDH3A1 whose high activity is prominent in several malignant tumors and highly expressed in the stomach, lung, keratinocytes, and cornea (33–35). In 2007, Ginestier and colleagues identified CD24CD44+ and ALDH+ cells in primary breast xenografts that displayed the greatest tumor-initiating capacity, generating tumors in NOD/SCID mice (21). Subsequently, these markers were reported to be expressed in CSCs from a wide variety of carcinomas, including those of the pancreas, colon, lung, ovary, and prostate gland (22, 36–39). CSCs are regarded as the main cause of the incidence and progression of cancer and the failure of clinical tumor treatment. Accumulating evidence suggests that CSCs consist of different sub-populations that can interconvert among different states. Overexpression of one or more transcription factors, activating *trans*-differentiation processes and even ALDH activity can drive the switch among CSCs and non-CSCs as well as between different subsets of CSCs. These adaptive strategies adopted by cells in response to different types of therapies as chemo and radiotherapy and must be considered in drawing new therapeutic approaches. Notably, ALDH1A1 and ALDH1A3 are CSCs markers in many tumors and overexpressed in resistant cancer cells. Moreover, these enzymes are involved into the processes that determine the switching from undifferentiated to differentiated states. Such processes are highly dynamic, and the fate of stem-like state can be decided by ALDH1A1 expression/activity in tumor cells, suggesting that this enzyme might represent a concrete novel target for cancer treatment (40). In addition, pathways such as RA, Notch, Wnt and TGF-β, may regulate ALDHs in CSCs at the transcriptional and post-translational level (Figure 1B). For instance, it has been proposed that Wnt cell signaling pathway regulates the ALDH1A1 gene expression through β-catenin/T-cell factor (TCF)-dependent transcription, promoting drug resistance and participates in maintaining the stemness capacities of CSCs (41). Transforming growth factor β (TGF-β) negatively downregulates ALDH through the protein Smad4 (42). Furthermore, ALDH activity might be post-translational regulated by the NOTCH signaling pathway, through the induction of SIRT2 expression that deacetylates ALDH1A1 on Lys-353 residue and therefore promoting breast cancer tumorigenesis and growth (43). Finally, ALDH could protect CSCs against radio and chemotherapy by maintaining ROS at low levels, metabolizing RA (44) (Figure 1B).

## Aldehyde Dehydrogenases in Response and Resistance to Therapies

Conventional anticancer therapies, such as chemo and radiotherapy, mainly act on target tumor tissues through the production of ROS, that, in turn, lead to oxidative stress and DNA, lipid and protein damage (45, 46). Cancer cells have an abnormal ROS homeostasis (47); if on one hand ROS promote tumorigenesis, on the other, exceeding ROS “threshold” is toxic and might trigger several cell death mechanisms including apoptosis, senescence and ferroptosis (48, 49). By interacting with lipids, ROS induce the peroxidation of fatty acids both in an enzymatic and non-enzymatic way, altering membrane permeability and promoting the formation of lipid hydroxides and other high reactive aldehydes such as 4-hydroxy-2-non-enal (4-HNE), acetaldehyde, malondialdehyde (MDA) and 4-hydroxy-2-hexenal (4-HHE) (50). The resulting macromolecular damage promotes the activation of a wide range of DNA damage repair pathways, including nucleotide excision repair (NER), base excision repair (BER), homologous recombination (HR), and ATR/ATM cell cycle checkpoints (51). To avoid further propagation of lipid peroxidation (LPO) and to attenuate oxidative stress, clearance of highly reactive lipid species is exerted by cancer cells through the activation of cellular antioxidant and free radical scavenging systems including ALDH enzymes (17). CSCs that overexpress ALDH showed lower levels of ROS compared to differentiated cancer cells (52) due to an increased NRF2-mediated expression of antioxidant enzymes as GPX3, SOD-2, and HO-1 (53). In breast cancer, high ALDH activity is associated with overexpression of poly (ADP-ribose) polymerase 1 (PARP1) and Olaparib resistance (54). In addition, ALDH1A1 and 3A1 enzymes can contribute to chemotherapy resistance through the direct metabolism of oxazophosphorine family drugs into non-toxic metabolites (25). Moreover, ALDH1A1 overexpression is associated with chemo-radio resistance in esophageal (55), breast (56), and mesothelioma (57) cancers. In lung cancer, ALDH1A1 expressing cells are resistant to epidermal growth factor receptor (EGFR) tyrosine kinase inhibitor gefitinib (58). In ovarian cancer, ALDH1A1 contributes to Olaparib resistance enhancing microhomology-mediated end joining (MMEJ) activity (59). ALDH3A1, instead, metabolizes aldehyde products of LPO and its expression can be affected by some hormones such as progesterone and cortisone (60). Basically, CSCs take advantage of ALDH detoxifying activity to reduce ROS and impair therapy efficacy (Figure 1B). However, ALDH-mediated ROS and endoplasmic reticulum (ER) stress reduction also affect antitumor immunity, limiting immunogenic cell death (ICD) and promoting the establishment of an immunosuppressive microenvironment (Figure 1C) (61, 62). Indeed, RA derived from ALDH-mediated retinal metabolism is involved in regulatory T cell (Treg) differentiation, survival and activation in TME (63). Treg cells are immune cells involved in maintenance of immunological self-tolerance and suppression; the immune tolerance induced by Treg can also impair anti-tumor immunity (64). RA is secreted by multiple cells in the TME (e.g., macrophages, dendritic cells, eosinophil, and epithelial cells); acting in a paracrine fashion, inducing ALDH isoenzymes expression in lymphocytes (e.g., ALDH1A1 and ALDH1A2). This led to further RA production, resulting in an increased Treg cell number, activity, and immune tolerance (65–67). In gastrointestinal tract, dendritic cells (DCs) and macrophages of the lamina propria and of the mesenteric lymph nodes express high levels of ALDH1A2 and ALDH1A1 and contributes to T cells differentiation (65, 67). Accordingly, in a murine model, co-colture of macrophages with native CD4^+^ T cells promote the activation of Treg cells in a RA/TGF-β/IL-10 dependent manner (68). In addition, macrophages release granulocyte–macrophage colony-stimulating factor (GM-CSF) that promotes ALDH expression in DCs (69). In lung tissue, TGF-β production and peroxisome proliferator-activated receptor γ (PPARγ) are crucial for the expression of ALDH1A2 and RA in DCs (70). Furthermore, PPARγ drives the expression of IL-6 and IL-23 in DCs, thus contributes to the maintainment of Treg function (66). In lung, macrophages expressing ALDH can promote differentiation of CD4^+^ T cells to Treg by TGF-β and RA (71). Hence, acting on ALDHs could revert these events and improve anti-tumor immunity (Figure 1C).

## Targeting Aldehyde Dehydrogenases in Cancer

Since ALDH overexpression is closely related to resistance to therapies, and to CSCs self-renewal, differentiation, and protection against oxidative stress, targeting these enzymes might represent a new potential strategy to overcome therapeutic resistance in cancer patients. Actually, ALDH inhibitors can be categorized on the basis of their specificity, in broad-spectrum ALDH inhibitors and in isoform-specific inhibitors. Such inhibitors are designed on the basis of crystallographic structures (72). Indeed, crystallographic studies have demonstrated that ALDH enzymes share high homology in the structure of three major domains: the NADP^+^-binding domain, the oligomerization domain, and the catalytic domain (73–75). The main differences between isoforms and what determines ligand specificity lay into the catalytic and the oligomerization domains (72). Such domains might be used for the development of highly selective molecules; instead, multiple ALDH inhibitors might be designed against the NADP^+^-binding domain, that is distinctive of ALDHs compared to other oxidoreductase enzymes (76). Another interesting approach to target ALDH enzymes is the use of 5-nitrofurans prodrugs to selectively target subpopulations of cell expressing high ALDH levels and displaying tumor initiating potential (77, 78). 5-nitrofurans are antibiotics widely used in veterinary medicine for the treatment of bacterial infections that in the last years have showed antitumor activity (79–81). Interestingly, it has been demonstrated that 5-nitrofurans, as nifurtimox and nifuroxazide, are bio-activated by ALDH1 and 2 enzymes into reactive nitro-species thus selectively target ALDH expressing melanoma stem cells, inhibiting tumor growth and initiation potential (78, 82). Furthermore, combination of nifurtimox and nifuroxazide with targeted therapy, as BRAF and MEK inhibitors, has demonstrate synergism *in vitro*, representing a potential new therapeutic approach in melanoma patients. In addition, a phase II clinical trial is currently undergoing on the use of nifurtimox in children with relapsed or refractory neuroblastoma or medulloblastoma^1^.

### Multi-Aldehyde Dehydrogenases Isoforms Inhibitors

Among the broad spectrum inhibitors, N,N-diethylaminobenzaldehyde (DEAB) is a commonly used competitive and reversible inhibitor provided as a negative control compound in the popular aldefluor assay (76). DEAB mainly acts on ALDH1A1 and 3A1 showing the inhibition of tumor growth and metastatic spreading in a mouse model of breast cancer (83). Another important group of multi-ALDH inhibitors is represented by the α,β-acetylenic amino thiolester family in which 4-dimethylamino-4-methyl–pent-2-ynthioic acid-S-methylester (DIMATE) is the most potent (84). DIMATE is an irreversible and competitive inhibitor of ALDH 1 and 3 (85). DIMATE has demonstrated to reduce tumor growth *in vitro* and when injected intraperitoneally in leukemia and melanoma models, showing also low toxicity on healthy cells (86, 87). In addition, an interventional clinical trial is active in France with the aim of investigate the effect DIMATE treatment on subpopulations of leukemic or normal stem cells^2^. No data are still reported from this trial. DIMATE has also been used in non-small cell lung cancer (NSCLC) models alone or in combination with cisplatin-based chemotherapy, causing the accumulation of aldehydes and aldehyde-protein adducts thus results in an increase of oxidative stress with no apparent toxic effects in treated animals (88). Despite this, no clinical trials and no oral bioavailability are still reported in literature. Other two inhibitors, dyclonine and aldi-6 have demonstrated tumor reduction in mouse models of head and neck squamous cell carcinoma (HNSCC) in combination with sulfasalazine or cisplatin, respectively (89, 90). Aldi-6 is administered to the mice through osmotic mini pumps in order to improve bioavailability and reduce dose, resulting in no mortality, no systemic toxicity and no body weight loss reported during the study (90). Recently, a novel series of potent multi-ALDH inhibitors has been identified through ligand-based docking studies (91). Among the inhibitors tested, the KS100 compound showed to be the most potent against ALDH1A1, 2, and 3A1, displaying anti-proliferative activity and increasing ROS levels and lipid peroxidation in several *in vitro* cancer models. However, KS100 showed toxicity *in vivo*, leading to a significant weight loss after 14 days of treatment (91). In order to reduce/overcome toxicity, researchers have developed a nanoliposomal formulation of KS100, called NanoKS100 (92). Such PEGylated liposomal formulation not only improved bioavailability, reducing spleen and liver accumulation but also demonstrating to be effective and not toxic even at high doses (92). Despite promising results, broad-spectrum ALDH inhibitors might retain toxicity due to the wide distribution of ALDH enzymes also in normal/healthy tissues (1). Nevertheless, the multi-ALDH inhibitors approach remains the most promising and effective. Several approaches in drug delivery systems are now under investigation in order to reduce toxicity, increase bioavailability and reducing off-targets effects in normal cells.

### Isoform-Specific Aldehyde Dehydrogenases Inhibitors

Isoform-specific inhibitors are under investigation in cancer research. Efforts have been made to identify new effective specific ALDH inhibitors for the main isoforms involved in cancer as ALDH1A1, 2, and 3A1 (72). Among ALDH1A1 selective inhibitors, a potent theophylline-based molecule, called NCT-501, has demonstrated *in vitro* efficacy on chemotherapy-resistant ovarian and HNSCC cell lines and reduction of tumor growth *in vivo* (93). This compound acts also on cisplatin-resistant models, with no toxicity. However, NCT-501 has limited bioavailability due to liver metabolism of the molecules before entry in systemic circulation (94). Furthermore, a novel ALDH1A family inhibitor named 673A has been reported to increase cell death in ovarian cancer promoting aldehyde-mediated DNA damage. The synergistic combination between 673A, that indirectly increases DNA damage, and ATM/ATR inhibitors, that impair DNA repair, promotes cell death, representing a potential new therapeutic strategy for ovarian cancer patients (95). Disulfiram represents a potent ALDH1A1 and ALDH2 inhibitor used for the treatment of alcoholism (96). *In vivo*, disulfiram is converted in its active metabolites, S-methyl-N,N-diethyldithiocarbamate (DETC) and S-methyl-N,N-diethyldithiocarbamate (Me-DDTC), two potent inhibitors of ALDH2 (97). Indeed, the addition of copper to disulfiram has been reported to enhance anti-tumor activity and reversing chemo-resistance in several drug-resistant cancer cells (98–100). A clinical phase 2 trial^3^ is ongoing on the use of Disulfiram combined with vinorelbine, cisplatin, and copper in metastatic hormone receptor positive, HER2 negative breast cancer patients. CB7 is an ALDH3A1 selective inhibitor that has been identified through an *in silico* docking-based high-throughput screening (101). This compound has demonstrated to be effective in combination with mafosfamide in lung cancer and glioblastoma *in vitro* (101). Another specific ALDH3A1 inhibitor is the represented by the reversible CB29 compound. However, this compound has demonstrated to have weak cytotoxicity on HNSCC models *in vitro* in combination with sulfasalazine (89). No *in vivo* studies are still ongoing with CB29. The main issues in using isoform-specific ALDH inhibitors lay in their low efficacy as monotherapy due mainly to the overlapping functions of ALDH family members. In fact, if one ALDH isoform is inhibited, another isoform take place, compensating its activity making also ineffective the treatment. Moreover, the use of an ALDH inhibitor could increase the ratio of effector T cells to Treg cells within tumor tissue leading to increased tumor immunity (63). For instance, inhibition of RA receptors has been shown to increase the efficacy of anti-tumor DC vaccines in a murine melanoma model through the suppression of tumor-infiltrating Treg cells and up-regulation of tumor-infiltrating, interferon-γ-secreting CD4+ and CD8+ T cells (102). Hence, early administration of an ALDH inhibitor in the context of combinatorial therapy could promote inhibition of the pro-tumor effect of Treg cells and enhancement of T-cell-mediated tumor rejection. Indeed, in a murine melanoma model, the administration of a PD-L1 and a-CTLA-4 combined with CSC-DC vaccine dramatically eliminated ALDH high CSCs following the triple combination treatment accompanied by a significantly enhanced T-cell expansion (103).

## Conclusions and Future Perspectives

Aldehyde dehydrogenases isoforms are often overexpressed by cancer cells, and, in particular, are responsible for the maintenance of the stem-cell-like phenotype, leading to cancer progression, chemo- and radio-therapy resistance, and immune evasion. Moreover, conventional and targeted therapies have demonstrated to induce/select cancer cells expressing high ALDH levels responsible of tumor recurrence and metastatic dissemination. Therefore, there is a growing interest in ALDH inhibitors for the treatment of cancer. Accordingly, numerous ALDH inhibitors have been developed, although many still lack clinical viability due to their toxicity, limited efficacy and/or bioavailability. However, researchers are developing several other inhibitors both isoform specific and with multiple targets focusing not only to the catalytic domain of ALDH. In the near future, research will be focused on less “famous” ALDH isoforms also. These own emerging important tumor-related functions, but the lack of crystallographic structure coupled with the absence of specific isoenzymatic assays has made the development of new effective and specific inhibitors difficult (72). Current available isoform-specific inhibitors have shown to be effective especially in combination. The main issue for these compounds remains the mild effect as monotherapy mainly due to the compensatory activity of ALDH enzymes that occurs when one isoform is inhibited (92). Multi-isoform ALDH inhibitors approach seems to be the most promising for translation into clinic. In addition, multi-ALDH inhibitors have been shown to be synergistic with several conventional treatments, such as chemo-, target- and radio-therapies for both inhibiting disease progression and preventing resistance development. Nevertheless, strategies to improve bioavailability and reduce toxicity are under study in preclinical models as new nano-formulation or device for drug delivery (90, 92). Such approaches have demonstrated to reduce off-target drug accumulation, improve both biodistribution and pharmacokinetic. ALDH inhibition could also increase the ratio of effector T cells to Treg cells within tumor tissue leading to anti-tumor immunity. Targeting ALDH activity affects also ALDH-expressing non-cancer cells of the TME, representing an intriguing new approach when combined with immunotherapy. Limiting ALDH activity and thus RA availability in the TME might impair differentiation and activation of Treg cells attenuates the immunosuppressive milieu. Coupling this with, for example, PD-1 or PD-L1 blockade may restore the activity of exhausted CD8 effector T cells inducing tumor rejection.

## Author Contributions

MZ and CA conceived and designed the review. MZ, SB, FF, and CA contributed to the drafting and figures and contributed to the manuscript revisions. All authors contributed to the article and approved the submitted version.

## Conflict of Interest

The authors declare that the research was conducted in the absence of any commercial or financial relationships that could be construed as a potential conflict of interest.

## Publisher’s Note

All claims expressed in this article are solely those of the authors and do not necessarily represent those of their affiliated organizations, or those of the publisher, the editors and the reviewers. Any product that may be evaluated in this article, or claim that may be made by its manufacturer, is not guaranteed or endorsed by the publisher.

## Footnotes

1
https://clinicaltrials.gov/ct2/show/results/NCT00601003

2
https://clinicaltrials.gov/ct2/show/NCT02748850

3
https://clinicaltrials.gov/ct2/show/NCT04265274

## Abbreviations

ALDHs: aldehyde dehydrogenases
DSB: DNA double strand breaks
ROS: reactive oxygen species
TME: tumor microenvironment
ICD: immunogenic cell death
Tregs: regulatory T cells
RA: retinoic acid
RAR α: retinoic acid receptor α
RXR: retinoic X receptor
ER α: estrogen receptor α
SCs: normal stem cells
CSCs: cancer stem cells
NER: nucleotide excision repair
BER: base excision repair
HR: homologous recombination
4-HNE: 4-hydroxy-2-non-enal
MDA: acetaldehyde malondialdehyde
4-HHE: 4-hydroxy-2-hexenal
LPO: lipid peroxidation
PARP1: poly (ADP-ribose) polymerase 1
EGFR: epidermal growth factor receptor
MMEJ: microhomology-mediated end joining
ER: endoplasmatic reticulum
DEAB: N,N-diethylaminobenzaldehyde
HNSCC: head and neck squamous cell carcinoma
DETC: S-methyl -N,N-diethyldithiocarbamate
Me-DDTC: S-methyl -N,N-diethyldithiocarbamate
TGF- β: transforming growth factor β
DCs: dendritic cells
GM-CSF: granulocyte–macrophage colony-stimulating factor
PPAR γ: peroxisome proliferator-activated receptor γ
DIMATE: 4-dimethylamino-4-methyl-pent-2-ynthioic acid-Smethylester.
